# One-stage Closure of the Small Non-growing Bladder Plate: New Insight into the Anatomy of Exstrophy - Trapezoid Interpubic Ligament (TIPL)

**DOI:** 10.1590/S1677-5538.IBJU.2025.0052

**Published:** 2025-05-20

**Authors:** Vasily V. Nikolaev, Nikita V. Demin

**Affiliations:** 1 Pirogov Russian National Research Medical University Moscow Russia Pirogov Russian National Research Medical University, Moscow, Russia; 2 Clinical and Research Institute of Emergency Pediatric Surgery and Trauma Moscow Russia Clinical and Research Institute of Emergency Pediatric Surgery and Trauma, Moscow, Russia

**Keywords:** Bladder Exstrophy, Exstrophy, Urogenital Abnormalities

## Abstract

**Purpose:**

The purpose of this study is to examine whether retrovesical fibromuscular structures—specifically the trapezoid interpubic ligament (TIPL)—mechanically restrict the inversion of small, non-growing bladder plates (SNGBP) in bladder exstrophy, and to evaluate bladder growth after one-stage closure with TIPL dissection, including the effect of anticholinergic therapy.

**Materials and Methods:**

Between 2004 and 2023, 15 patients with SNGBP underwent one-stage bladder closure using a modified surgical approach with TIPL dissection. The TIPL, identified as a fibromuscular structure impeding bladder plate (BP) inversion, was targeted. Postoperative bladder capacity was evaluated based on age at surgery and the use of anticholinergic therapy.

**Results:**

The TIPL was identified as the primary mechanical impediment to BP inversion. Its dissection restored tissue compliance, facilitating successful one-stage closure in all patients. In children under three years of age at the time of surgery, the mean annual bladder capacity increased by 17.76 mL. Anticholinergic therapy further enhanced bladder growth.

**Conclusion:**

TIPL dissection enables one-stage closure in SNGBP patients who were previously considered unsuitable for this method. Early intervention supports bladder development and favorable functional outcomes. These findings provide novel anatomical insights, warranting further morphological and embryological research to validate the universality of this structure and technique.

## INTRODUCTION

Bladder exstrophy, a rare and complex congenital anomaly, severely alters the lower urinary tract, anterior abdominal wall, and pelvic ring anatomy. Despite notable progress in surgical reconstruction in recent decades, various anatomical and biomechanical factors, especially those affecting primary bladder closure feasibility, remain inadequately elucidated (1–5). Patients with small BPs, typically characterized by a width under 3 cm and inadequate tissue for tension-free closure, pose a significant challenge (6). Primary closure is often deemed unfeasible due to suboptimal tissue quality, heightened stiffness, and limited growth capacity. Closure feasibility is assessed under anesthesia via the BP inversion test, applying gentle pressure to evaluate compliance. Failure to invert the plate is generally regarded as a contraindication to primary closure (7, 8). In such cases, a common approach is watchful waiting to permit spontaneous growth and improved compliance (6). If growth does not occur, cystectomy with enterocystoplasty is typically conducted (9). Alternatively, a two-stage method entails suturing the BP to the anterior abdominal wall in the neonatal period, creating a hernia-like sac to promote expansion via intra-abdominal pressure, though its advantage over natural growth lacks confirmation (10).

Among patients with small BP, a distinct subgroup exhibits no growth despite prolonged observation. These patients often wait years for BP expansion that, for reasons yet unclear, fails to materialize. Termed SNGBP, this subgroup constitutes roughly 10% of classical bladder exstrophy cases. While resistance to BP inversion critically affects surgical decisions, its anatomical and histological underpinnings remain elusive. Fibrotic remodeling is frequently presumed to be the chief constraint on tissue pliability. We hypothesize that, beyond intravesical fibrosis, the biomechanical attributes of retrovesical fibromuscular structures in the interpubic region may serve as an additional mechanical impediment, limiting BP inversion and decreasing the probability of successful primary closure. To test this hypothesis, we defined two objectives:

- To investigate whether retrovesical fibromuscular structures mechanically restrict bladder plate inversion, thus impeding primary closure in patients with SNGBP.
- If mitigating their mechanical effect facilitates closure, to analyze bladder growth dynamics and their reliance on anticholinergic therapy.

## MATERIALS AND METHODS

### Patient Selection

This cohort study, conducted from 2004 to 2023, encompassed all patients referred to our institutions with a diagnosis of bladder exstrophy accompanied by a SNGBP. Initially, patients were admitted to various neonatal surgical departments shortly after birth. These individuals were deemed unsuitable candidates for bladder closure due to inadequate bladder elasticity, diminished bladder size, multiple hamartomatous polyps, and unfavorable findings during examination under anesthesia (which assessed the feasibility of BP inversion into the pelvic cavity). The inclusion criteria comprised absence of prior surgical correction of bladder exstrophy, age exceeding six months, and a non-invertible BP measuring less than 3 cm.

### Ethics

This study was approved by the research ethics committees of both participating institutions (No. 05/1602 and No. 180226).

### Preoperative Examination

Pelvic MRI was conducted in recent cases before surgery to identify anatomical structures potentially limiting BP inversion and to correlate MRI findings with intraoperative observations. This method assessed MRI's diagnostic utility in preoperative planning and explored the visibility of the interpubic ligamentous complex.

### Surgical Procedure

All patients underwent uniform BP closure, adapting a previously reported technique (11) for this study. Modifications included microdissection of retrovesical structures obstructing SNGBP inversion. Large BP polyps hindering closure were excised in 4 of 15 patients. The bladder was inverted, sutured longitudinally, and shaped with a funnel-like neck over a No. 8Ch pigtail catheter, then placed in the pelvic cavity. Ureteral reimplantation was not performed during primary closure. Paravesical fat from the Retzius space was mobilized from the pelvic walls and sutured anteriorly to the bladder neck. Microdissection utilized surgical loupes (×3.5 to ×6.5 magnification), a monopolar microdissection needle electrode (fine tip), and microsurgical scissors. Among the 15 patients, pubic bone approximation was performed in 7 more recently treated cases.

A bone-holding clamp reduced interpubic diastasis, and fixation was achieved with two opposing U-shaped interrupted No. 2 Vicryl sutures. This was indicated when the interpubic gap surpassed 4 cm or pelvic instability persisted post-bladder plate positioning.

### Postoperative Management

All patients received intravenous broad-spectrum antibiotics from surgery until discharge, followed by oral prophylaxis. Oxybutynin anticholinergic therapy (0.4 mg/kg/day) began on the first postoperative day and continued until bladder catheter removal. Patients rested in bed or their mother's arms without immobilization and were discharged between postoperative days 8 and 17 (median: 11.6 days). Per protocol, boys underwent epispadias repair 6–12 months later (12).

### Outcomes Assessment

Successful bladder closure was defined as an intact repair showing no bladder prolapse, dehiscence, vesicocutaneous fistula, or outlet obstruction (13). For patients over 1 year, bladder capacity monitoring involved parental measurement of morning urine volume, routine ultrasound, and voiding video documentation. Per protocol, evaluations occurred at 1, 3, and 5 years, and prior to bladder neck reconstruction. In delayed primary closure cases, study timing was tailored to clinical status. In-hospital capacity was assessed via gravitational cystometry during cystography under anesthesia, maintaining physiological pressure at 15 cm H2O. Patients over five years with bladder capacity above 60 mL underwent subsequent bladder neck reconstruction. Continence was assessed in children at least six years old, typically one-year post-bladder neck reconstruction (BNR). Urinary continence was defined as a dry interval of 3+ hours without nocturnal enuresis (14). Follow-up spanned 12 months to 9 years (median: 4.6 years). No patients were lost to follow-up, with ongoing communication sustained via online contact with one author (VVN). Families regularly reported voiding patterns, outpatient ultrasound findings, and urinalysis results.

## Statistical Methods

We statistically analyzed bladder capacity increases with and without anticholinergic agents using the R programming language. The difference between therapy initiation and volume measurements was determined, dividing patients into "before" and "after" administration groups. The ggplot2 library facilitated visualization of bladder capacity changes and range diagrams. To evaluate differences in capacity increase rates pre- and post-anticholinergic therapy, we computed the rate of increase between measurements. A matched-pairs Wilcoxon test was used to compare the groups. Medians and quartiles were calculated as descriptive statistics (15).

## RESULTS

### Management before referral

Seven patients in the cohort received botulinum toxin injections into the detrusor muscle at their neonatal centers to enhance BP compliance, yet subsequent anesthesia-based assessments revealed no measurable improvement. These non-invertible cases were referred to our center for further evaluation and surgical intervention.

### Primary Bladder Closure

Fifteen patients, aged 6 to 85 months (including three girls), underwent a modified primary bladder closure procedure. Enterocystoplasty was performed in one 5-year-old female with an extremely small (<2 cm), polyposis SNGBP, where primary closure was considered unfeasible.

### TIPL Dissection and Bladder Plate Inversion

**Figure 1:**
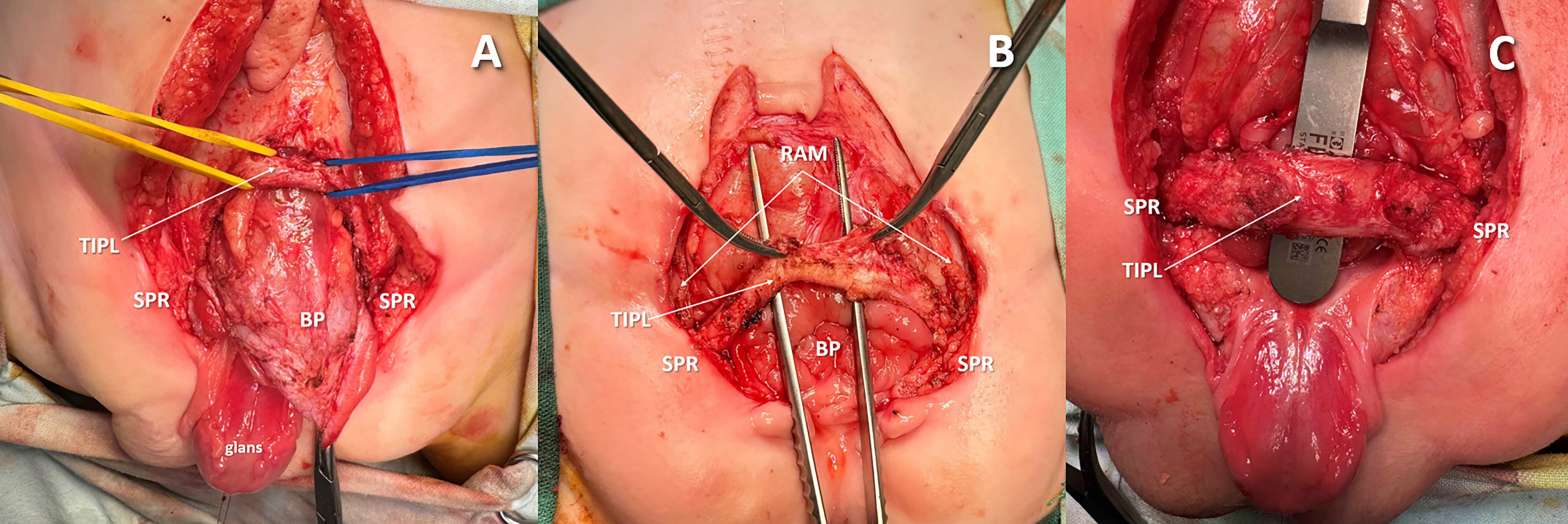
Separation of the TIPL from the detrusor.

**Figure 2:**
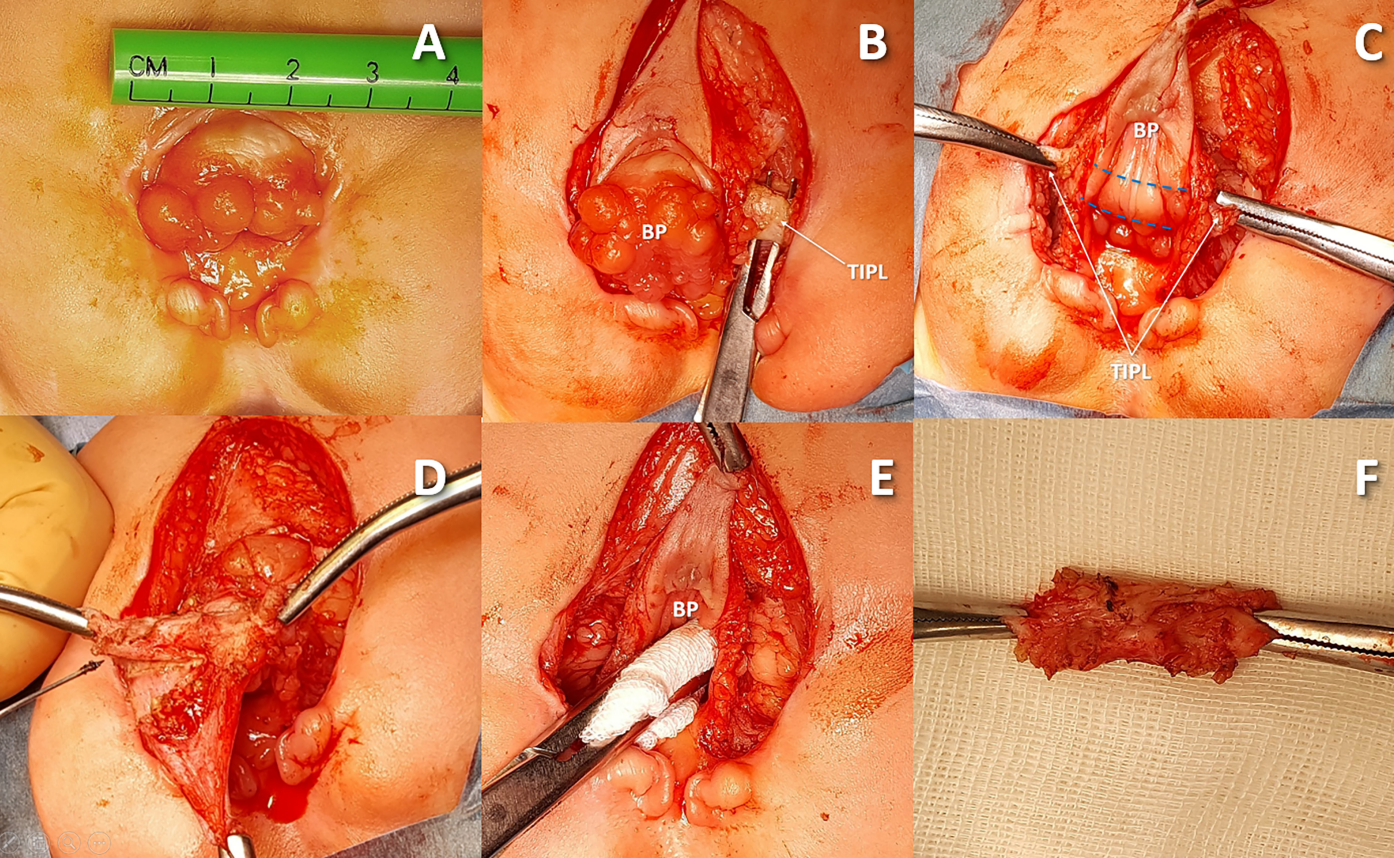
Stages of dissection and small bladder plate compliance restoration through complete dissection of the TIPL.

**Figure 3:**
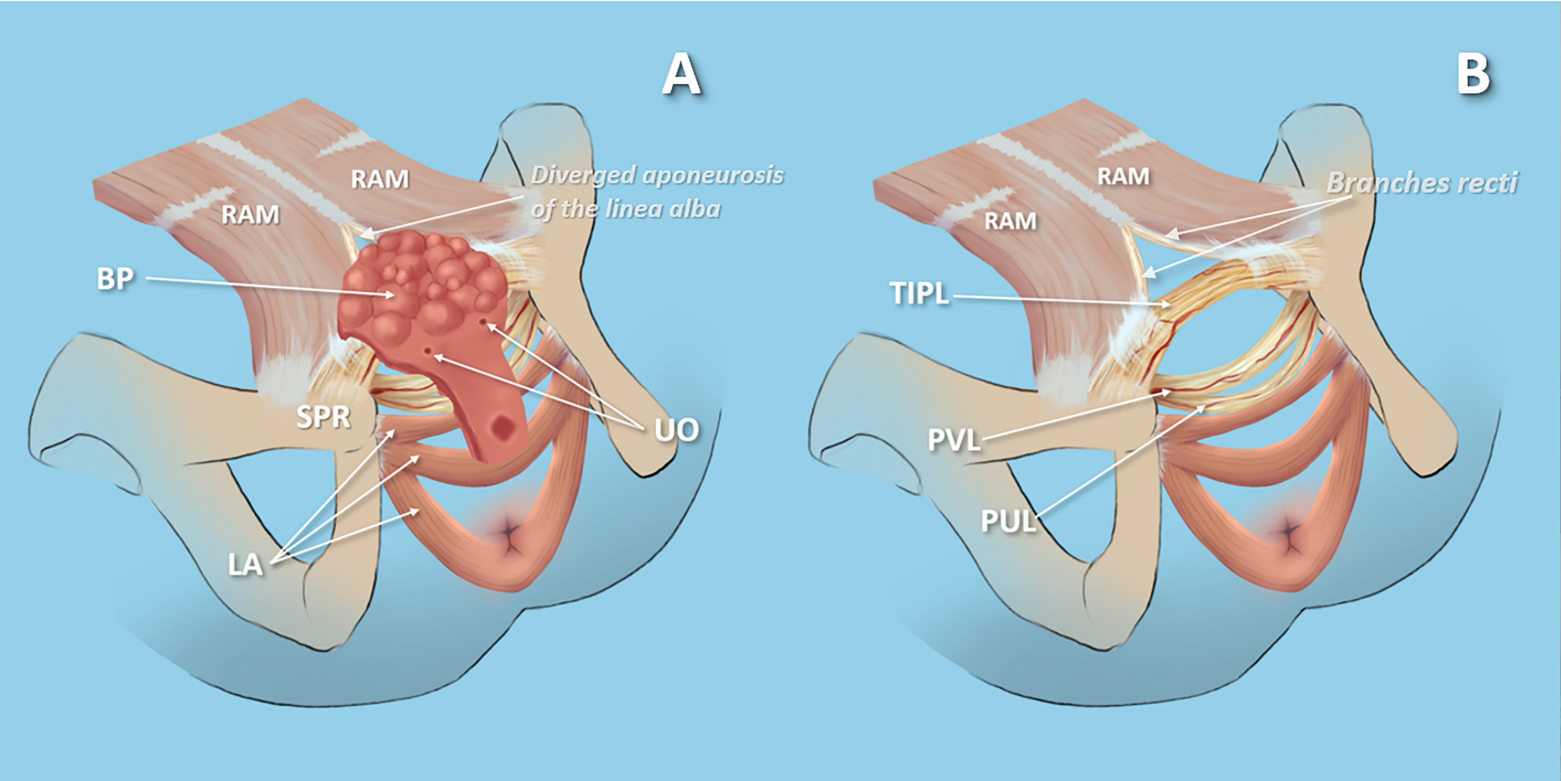
Anatomical disposition of the trapezoid interpubic ligament (TIPL).

Intraoperative exploration identified a previously unreported fibromuscular structure, tightly linked to the BP and impeding its inversion in all SNGBP patients. Named the Trapezoid Interpubic Ligament (TIPL) (Figure-1), this ligament consistently restricted BP inversion. It was securely anchored to the detrusor in the supratrigonal region and to the diverging aponeurotic fibers of the linea alba, forming the medial boundaries of the rectus abdominis muscle (RAM) sheaths. Cranio-lateral traction from the RAM through these fibers shaped the ligament's distinctive trapezoidal form. Intraoperative findings revealed that transecting the TIPL near the pubic bones alone did not enable BP inversion. When the ligament remained attached to the detrusor, inversion was unachievable despite partial release. Inversion occurred only after complete detachment of the TIPL from the detrusor (Figure-2). To clarify the TIPL's relationship with adjacent pelvic ligaments, targeted anatomical dissection was performed in six patients. The ligament was separated from the detrusor and rectus abdominis sheaths while preserving its pubic bone attachment. Detaching the TIPL from the rectus sheath relaxed the ligament and consistently widened the interpubic gap, mirroring effects seen post-transection. Bladder plate inversion required full detachment from the detrusor. Near its attachment to the superior pubic rami, the pubovesical (PVL) and pubourethral (PUL) ligaments were inserted into the TIPL at a 70–90° angle and were transected there to facilitate deep pelvic descent of the vesicourethral segment. The PVL ran nearly horizontally under the trigone, while the PUL extended more caudally beneath the proximal urethral plate. After deeper descent of the bladder plate, the TIPL, remaining in its original position, came to lie anterior to it (Figures 1B and C). Figure-3 illustrates the spatial relationships among the TIPL, PVL, PUL, and BP relative to the anterior abdominal wall defect.

**A)** from RAM and bladder plate **B, C.**

**A)** A horizontal part of TIPL at the RAM diastasis (between the holders) following the separation of the ligament from the detrusor, but without the dissection from the RAM sheath (BP turned caudally). **B, C)** TIPL is isolated from the RAM sheath and the bladder plate with preserved attachment to the SPR.

**A)** Small bladder plate in a 30-month-old girl. **B)** Initial stages of ligament separation. Clamp is placed underneath the TIPL between the pubic bone and the bladder plate. **C)** TIPL is dissected from the pubic bones, but remains attached to the detrusor. At this stage, the bladder plate remains non-invertible, highlighting the importance of detrusor separation and division of pubic ligamentous attachments. **D)** Technique of TIPL separation from the detrusor muscle. **E)** Free inversion of the urinary bladder with a gauze ball following the dissection of the retrovesical ligament from the detrusor. **F)** Gross TIPL preparation following separation from the pubic bones, detrusor and the RAM sheath.

**A)** Diagram illustrating the SNGBP and anatomical structures surrounding the bladder plate and the infraumbilical anterior abdominal wall defect. **B)** Position of the TIPL, pubovesical, and pubourethral ligaments in the absence of the bladder plate. Attachments of the TIPL to the diverging aponeurosis of the linea alba and the rectus abdominis sheaths deform the ligament, pulling it cranially and giving it a trapezoid shape.

## MRI study

**Figure 4:**
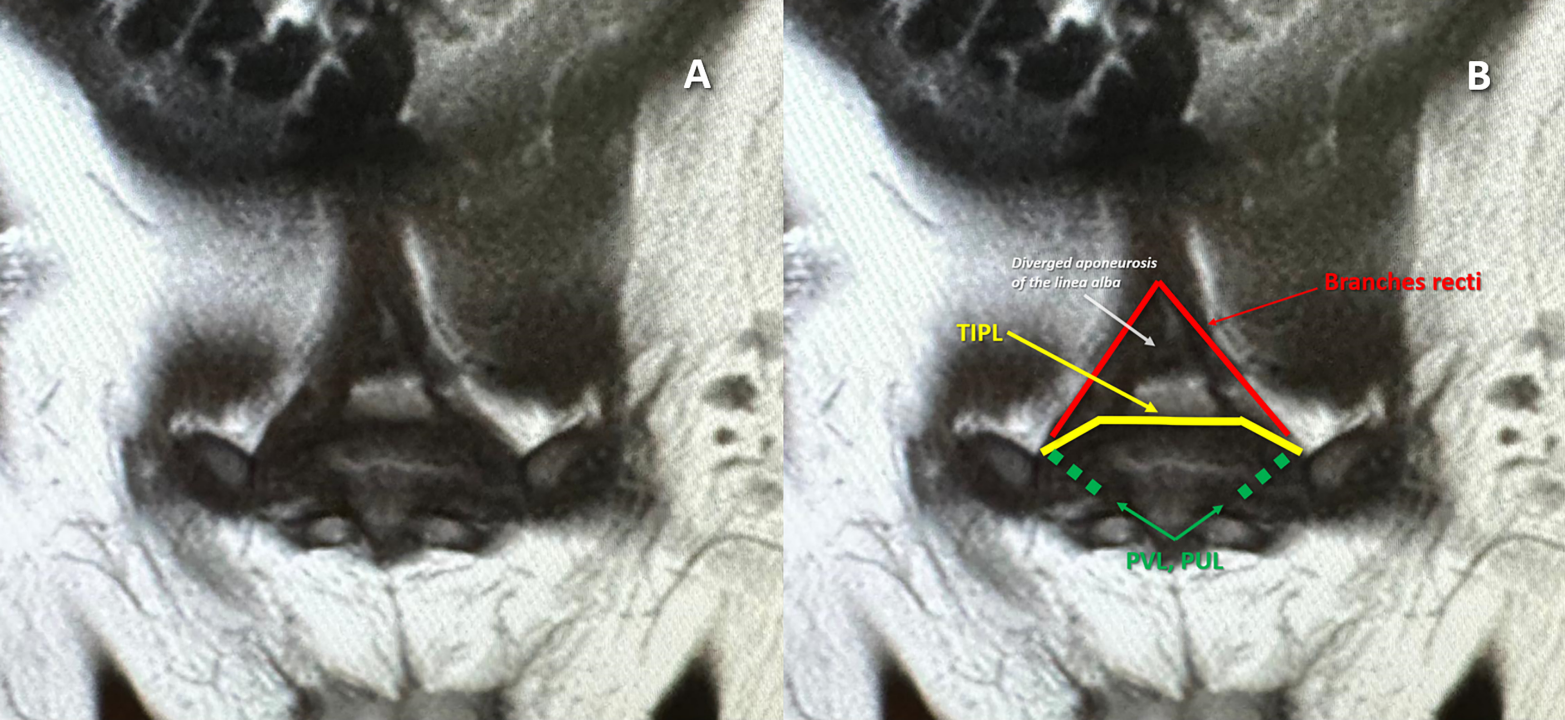
MRI image of SNGBP.

Pelvic MRI, conducted in five patients from the cohort's latest subset, revealed a trapezoid-shaped structure spanning from the supratrigonal region to the medial edges of the rectus abdominis muscles in all cases. Its oblique alignment and partial overlap with adjacent tissues prevented MRI from fully defining its continuity or attachments. These observations were correlated with intraoperative findings to evaluate MRI's diagnostic utility in preoperative planning (Figure-4).

**A, B)** The image clearly demonstrates divergence of the linea alba aponeurosis (red) and its fusion with the Trapezoid Interpubic Ligament (TIPL, yellow). The directions of the pubourethral (PUL) and pubovesical (PVL) ligaments are marked in green but are less distinct due to the mismatch in imaging planes.

## Biopsy

**Figure 5:**
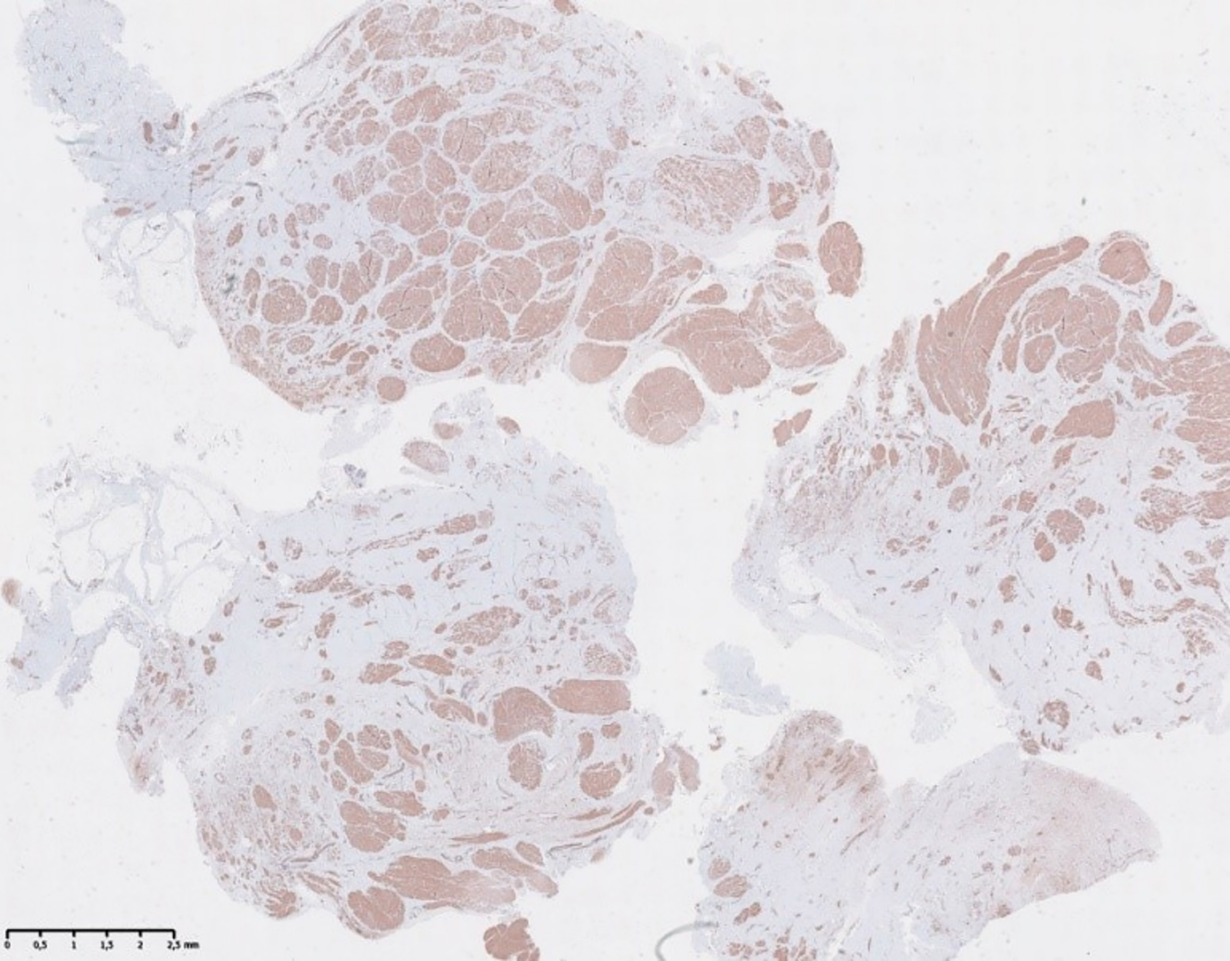
Microscopic TIPL preparation.

Gross examination of the TIPL revealed a round or elliptical 5–9 mm cross-section in most patients, with greater thickness in those with a narrower interpubic space. In one case, the TIPL formed a 20 mm wide, 12 mm long, 5 mm thick plate, anchored to the pubic bones by paired short filaments. Biopsies from the midportion of the TIPL in 10 patients showed a fibromuscular composition with plentiful smooth muscle bundles, consistent with a ligament (Figure-5).

Reaction with SMA (smooth muscle actin) - smooth muscle bundles. Collagenic fibrous tissue with few cells and incorporated smooth muscle bundles.

### Clinical Outcomes and Bladder Growth

Bladder closure was successfully completed in all 15 cases. Three patients had minor skin dehiscence (Clavien-Dindo I) anterior to the neomeatus. One developed a urethrocutaneous fistula (Clavien-Dindo II) that healed spontaneously. Two experienced febrile urinary tract infections from transient bladder neck obstruction post-catheter removal, effectively treated with urethral stenting and antibiotics.

### Bladder Capacity and Continence Outcomes

Patients undergoing bladder closure before age three exhibited notable bladder capacity gains, with a mean annual increase of 17.76 mL/year. Anticholinergic agents further enhanced growth. Pre-therapy, the median monthly bladder capacity increase was 1.48 mL/month (1st quartile: 0.89; mean: 1.90; 3rd quartile: 2.50), rising to 2.63 mL/month post-therapy (1st quartile: 1.75; mean: 2.64; 3rd quartile: 3.52). A matched-pairs Wilcoxon test showed no significant difference in growth rates pre- and post-therapy (p = 0.4469), though visual trends indicated increased growth post-therapy. Bladder neck reconstruction (BNR) occurred in 8 of 9 patients over five with capacities above 60 mL, yielding positive continence results, with most achieving daytime continence and some full continence without nocturnal enuresis (Table-1).

**Table 1:** Patients who underwent bladder closure, the surgery performed and the increase in bladder capacity.

N° of patients/sex/age (month) of BC	1 y/o	3 y/o	5 y/o	7 y/o	12 y/o	Results of BNR (3-8y/o outcome	Pubic bones approximation
1/m/8m	11	17	44>	>	162BNR	Continence	-
2/f/30m	0	12>	75/BNR			Continence	-
3/m/10m	10	38	47>	110/BNR		Day continence	-
4/f/85m	0	0	0	BC>	30BA+ECP		-
5/m/7m	22>	70 ☒	95/BNR			Day continence	-
6/m/13m	0	34>	52>	83/BNR		Day continence	-
7/m/6m	18>	86 ☒	113/BNR			Partial continence 0.5-1h	-
8/m/8m	15	29>	65/BNR			Partial continence 0,5-1,0-1h	-
9/f/13m>	0	92 ☒	130/BNR			Day continence	+
10/m/6m>	38>	141 ☒					+
11/m/36m>	0	>	51>				+
12/m/11m>	6>	33>					+
13/m/14m>	0	80 ☒					+
14/m/7m	17>						+
15/m/6m>	20>						+

f = female; m - male

> start or continuation of longstanding cholinolytic use X -discontinuation of cholinolytics BC - Bladder Closure

BA = Bladder augmentation

BNR = Bladder Neck Reconstruction

## DISCUSSION

Reconstructive surgery for exstrophy fundamentally aims to preserve all normal lower urinary tract anatomical structures (16, 17). However, reconstruction is only feasible after transection of the ligaments that anchor the bladder plate to the pelvic sidewalls. Historically, exstrophy was thought to lack normal pubic ligaments (anterior, posterior, superior, and inferior) (18, 19). Wood (1869) described a robust fascial membrane connecting the PB behind the urethra, supporting the bladder base and penis, yet noted no interpubic structures above or anterior to the urethra (20). Shattock (1994), examining St. Thomas’ Hospital Museum specimens, confirmed the bladder muscle wall's attachment to the PB's posterior surface, identifying the suburethral filamentous triangular urethral ligament (urogenital diaphragm) as the sole fibrous PB connection in exstrophy (21). Mid-20th-century surgeons developing systematic reconstruction also found no anterior pubic ligaments (16), reinforcing the view that pubic ligaments are absent in exstrophy, with lateral bladder attachments tied to urogenital diaphragm structures (22). The surgical community maintained this view largely unquestioned until the modern era.

Before microdissection, we, like other surgeons, assumed the short segment between the BP and superior pubic ramus was the pubovesical ligament (23, 24). Microdissection, however, clarified that this is merely a component of the TIPL, affiliated not with the urogenital diaphragm but with the anterior abdominal wall. The TIPL's lateral portions merge with the inner aponeurotic edges of the rectus abdominis sheaths, resembling a divided linea alba aponeurosis—characteristic of the anterior pubic ligament. This supports the view that the TIPL originates from and integrates with anterior abdominal wall structures, aligning with the anterior pubic ligament's configuration, not the urogenital diaphragm as previously thought, potentially explaining its absence from earlier anatomical descriptions (19, 25, 26).

This explains why the TIPL remained unidentified. Standard BP mobilization begins with detachment from the rectus abdominis sheaths for access, followed by severing all connective structures between the BP and pubic bones, typically through an incision along the superior pubic ramus. Alternatively, surgery starts at the pubic tubercles, promptly transecting ligaments to the pubic bones, then accessing the paravesical space via Retzius fat, and separating the BP from fascial layers and peritoneum. These techniques prioritize surgical efficiency and safe mobilization over preserving ligamentous anatomy, obscuring original insertions and interpubic connections before identification. Moreover, fixed formalin specimens apt for detailed dissection are scarce, and post-fixation dissection of fused fibromuscular structures is markedly challenging. Thus, prior studies’ failure to note the TIPL likely stems from its routine transection, not its absence.

Though the BP in SNGBP cases is often labeled inelastic or fibrotic, our findings suggest its limited pliability largely results from extrinsic mechanical traction by the TIPL. Full dissection from the detrusor considerably enhanced compliance in all cases, enabling inversion. This implies that reduced elasticity is partly mechanically induced and reversible, not solely due to intrinsic fibrosis.

Near its pubic attachment, the pubovesical (PVL) ligaments join the TIPL at a 70–90-degree angle, while the pubourethral (PUL) ligaments merge slightly more caudally and laterally. Typically, PVL and PUL insert into the posterior pubic ligament (PPL), as supported by studies of the normal pubic symphysis (19, 25, 26). Pieroh et al. (2021) identified the PPL as a distinct ligament reinforcing the pubic periosteum, linking to lateral PVL fibers and receiving PUL terminal insertions. These findings reveal the TIPL, by integrating PVL and PUL, mirrors the PPL's topographical and morphological traits (19), suggesting it may be a persistent or unregressed element of this ligamentous complex in the exstrophic pelvis. Collectively, these observations suggest that the TIPL is a fibromuscular complex that topographically and structurally combines traits of all known pubic ligaments—both anterior and posterior—into a single, previously unrecognized interpubic entity in exstrophy cases.

The widened interpubic space following ligament mobilization and transection highlight its role in maintaining pelvic anatomical stability. Though seemingly paradoxical, this aligns with the ‘posterior’ designation of the posterior pubic ligament, which pertains to its position relative to the pubic bones, not the bladder. In exstrophy's everted anatomy, this ligamentous structure shifts behind the BP, whereas in normal anatomy, it would reside anterior to the bladder and posterior to the pubic symphysis.

Though fibromuscular like the urogenital diaphragm or pelvic floor, we propose the TIPL as a unique anatomical structure. Pre-inversion, it lies posterior to the BP within the interpubic space, arising from the pubic bone periosteum and extending cranio-medially into the rectus abdominis sheath—unlike the caudal, dorsal orientation of pelvic diaphragm muscles. Histologically, the predominance of smooth muscle bundles indicates a visceral rather than skeletal origin, distinguishing the TIPL from pelvic floor structures such as the voluntary urethral sphincter and the levator ani. We posit that the TIPL is a persistent midline mesenchymal remnant of the anterior abdominal wall, unregressed from embryogenesis, mechanically impeding BP inversion in exstrophy. This sets it apart from the dynamic muscular supports tied to continence in prior studies (27).

Clinical data revealed a mean bladder capacity increase of 17.76 mL/year without anticholinergic therapy, exceeding prior reports (6–14 mL/year) (28, 29). Anticholinergic use further boosted growth. We attribute this to enhanced detrusor compliance post-TIPL dissection and the lack of PB approximation in long-term cohort cases, as bone approximation reduces pelvic volume and may restrict bladder expansion. Closure before age three correlated with normal bladder growth. Eight of ten patients showed rising capacity, facilitating later bladder neck reconstruction with positive outcomes. These findings are promising, given SNGBP patients’ complexity within the exstrophy spectrum. Despite low initial capacity, their growth neared normal rates (~18 mL/year vs. ~30 mL/year in healthy children), with minimal surgical complications. One patient underwent delayed bladder closure at age 7, followed by enterocystoplasty at 12 due to insufficient growth, highlighting the limitations of extended observation in SNGBP and the risk of missing optimal functional development windows. This supports early primary closure once mechanical barriers like the TIPL are resolved.

To the best of our knowledge, this is the first documented successful one-stage closure in an SNGBP cohort, a subgroup historically deemed unfit for this approach. Though small, this rare cohort reflects years of observation of an underexplored population. We identified a novel fibromuscular structure, likely a persistent pubic ligament bundle, previously thought absent in exstrophy. Its smooth muscle fibers suggest an immature phenotype tied to failed pubic fusion and persistent diastasis. The structure's anatomy and location imply incomplete regression of ventral mesenchymal elements during embryogenesis. These insights may extend beyond SNGBP, enhancing understanding of ventral body wall defects and informing surgical and developmental models of lower abdominal wall formation, aligning with recent embryological research on midline fusion complexities (30).

Despite the importance of identifying the TIPL, this study faces limitations. The small sample size limits the findings’ generalizability. The extended study duration introduces variability in patient care and compromises data uniformity. Furthermore, incomplete data on TIPL variations across BP sizes hinder confirmation of result universality. MRI visualization of TIPL awaits further validation. While no surgical complications arose from TIPL dissection, its proximity to the detrusor demands precision to avoid impairing BP vascularity. Currently, no publications detail technique modifications for one-stage SNGBP closure, precluding comparisons with our approach.

## CONCLUSION

In conclusion, identifying and defining the TIPL as a fibromuscular complex obstructing SNGBP inversion enhance our understanding of exstrophy anatomy. This enables one-stage closure of small bladder plates, fosters bladder growth, and enhances functional outcomes. Further research integrating embryological, morphological, and clinical data is essential to validate this technique's universality and optimize surgical methods. Though small, this cohort represents a rare, clinically significant bladder exstrophy subgroup—patients with small, SNGBP. The condition's scarcity has impeded anatomical study and surgical progress, rendering even modest data impactful. Our findings offer novel anatomical insights that could shape future surgical approaches for this challenging population.

## ABBREVIATIONS

BP: = Bladder plate
SNGBP: = Small non-growing bladder plate
TIPL: = Trapezoid Interpubic Ligament
PVL: = Pubovesical ligament
PUL: = Pubourethral ligament
RAM: = Recti abdominis muscles
PB: = Pubic bones
SPR: = Superior pubic ramus
BC: = Bladder Closure
BNR: = Bladder Neck Reconstruction
